# High-Resolution DWI with Simultaneous Multi-Slice Readout-Segmented Echo Planar Imaging for the Evaluation of Malignant and Benign Breast Lesions

**DOI:** 10.3390/diagnostics11122273

**Published:** 2021-12-04

**Authors:** Shuyi Peng, Yihao Guo, Xiaoyong Zhang, Juan Tao, Jie Liu, Wenying Zhu, Leqing Chen, Fan Yang

**Affiliations:** 1 Department of Radiology, Union Hospital, Tongji Medical College, Huazhong University of Science and Technology, Wuhan 430022, China; shuyipeng@hust.edu.cn (S.P.); 2014xh0917@hust.edu.cn (J.T.); liu_jie0823@163.com (J.L.); zhuwenying917@163.com (W.Z.); leqingchen@hust.edu.cn (L.C.); 2 Hubei Province Key Laboratory of Molecular Imaging, Wuhan 430022, China; 3 MR Collaboration, Siemens Healthcare Ltd., Guangzhou 510645, China; yihao.guo@siemens-healthineers.com; 4 MR Collaboration, Siemens Healthcare Ltd., Shenzhen 518057, China; zhang.xiaoyong@siemens-healthineers.com

**Keywords:** diffusion-weighted imaging, breast, simultaneous multi-slice acceleration, readout-segmented echo planar imaging

## Abstract

To investigate the feasibility and effectiveness of high-resolution readout-segmented echo planar imaging (rs-EPI), diffusion-weighted imaging (DWI) is used simultaneously with multi-slice (SMS) imaging (SMS rs-EPI) for the differentiation of breast malignant and benign lesions in comparison to conventional rs-EPI on a 3T MR scanner. A total of 102 patients with 113 breast lesions underwent bilateral breast MRI using a prototype SMS rs-EPI sequence and a conventional rs-EPI sequence. Subjective image quality was assessed using a 5-point Likert scale (1 = poor, 5 = excellent). Signal-to-noise ratio (SNR), lesion contrast-to-noise ratio (CNR) and apparent diffusion coefficients (ADC) value of the lesion were measured for comparison. Receiver operating characteristic curve analysis was performed to evaluate the diagnosis performance of ADC, and the corresponding area under curve (AUC) was calculated. The image quality scores in anatomic distortion, lesion conspicuity, sharpness of anatomical details and overall image quality of SMS rs-EPI were significantly higher than those of conventional rs-EPI. CNR was enhanced in the high-resolution SMS rs-EPI acquisition (6.48 ± 1.71 vs. 4.23 ± 1.49; *p* < 0.001). The mean ADC value was comparable in SMS rs-EPI and conventional rs-EPI (benign 1.45 × 10^−3^ vs. 1.43 × 10^−3^ mm^2^/s, *p* = 0.702; malignant 0.91 × 10^−3^ vs. 0.89 × 10^−3^ mm^2^/s, *p* = 0.076). The AUC was 0.957 in SMS rs-EPI and 0.983 in conventional rs-EPI. SMS rs-EPI technique allows for higher spatial resolution and slight reduction of scan time in comparison to conventional rs-EPI, which has potential for better differentiation between malignant and benign lesions of the breast.

## 1. Introduction

Breast cancer is one of the most common malignancies among women in the world [1]. Magnetic resonance imaging (MRI) has unique advantages in the diagnosis and evaluation of breast lesions. It not only performs high accuracy in the detection and diagnosis of breast cancer, but it is also used to evaluate the treatment response of tumors through the information of pharmacokinetics, tissue microcirculation, and pathophysiology from a microscopic point of view [2,3]. Dynamic contrast enhancement magnetic resonance imaging (DCE-MRI) is the backbone scanning sequence of current breast MR, according to the American College of Radiology criteria [4]. In DCE-MRI, high resolution morphological information and semi-quantitative tumor angiogenesis characteristics can be provided using the dynamic changes of T1 signals after injection of gadolinium-based contrast agents [5]. The detection sensitivity of DCE-MRI of the breast on the 3T MRI scanner was nearly up to 100%, while specificity ranged from 49.1% to 87.5% [6,7,8,9]. To overcome the limitations of specificity, there is increasing interest in exploring the combined application of other functional parametric maps for differential diagnosis.

Diffusion-weighted imaging (DWI) that is able to detect the diffusion of water molecules in vivo to extrapolate and analyze tissue structure and internal characteristics has been proven to be a useful technique for the evaluation of breast cancer [6,10,11]. Previous studies [12,13] have demonstrated that DWI can provide additional functional information to DCE-MRI, and the combination of DCE-MRI with DWI yields a higher specificity (75–84%) versus DCE-MRI alone (67–72%). Moreover, the apparent diffusion coefficients (ADC) value derived from DWI can provide information for evaluating the treatment response to neoadjuvant chemotherapy [14,15,16]. For the DWI sequence, the most commonly used readout method is single-shot echo planar imaging (ss-EPI) in the current clinical standard because of its low sensitivity to motion-induced phase errors [17]. However, ss-EPI has limitations of strong susceptibility artifacts, geometric distortions, and poor spatial resolution due to a shorter T2* relaxation time, especially in the prepectoral region of the breast [17,18,19]. Furthermore, spatial resolution is limited by signal blurring in the phase-encoding direction.

To overcome the above two limitations (distortions and blurring) and improve the image quality of DWI, readout-segmented echo planar imaging (rs-EPI) that divides k-space into separate segments in the readout direction is proposed. A previous study [20] demonstrated that rs-EPI can reduce geometric distortions and blurring arising from T2* decay in DWI. Recent studies on breast also indicated that rs-EPI reduces geometric distortions and shows better image quality and lesion conspicuity, compared to ss-EPI [17,20,21]. However, rs-EPI requires long acquisition time that may adversely affect its use in clinics. and the reduction of the acquisition time may have a negative impact on image quality, particularly in the high-resolution acquisition that is critical to discriminate between heterogeneous tumor regions or between tumor and normal tissue. Recently, simultaneous multi-slice (SMS) acquisition based on the blipped ‘controlled aliasing in parallel imaging results in higher acceleration’ (blipped CAIPIRINHA) technique, which can be used to achieve spatial shifts in the phase-encoded direction between simultaneously excited slices, has been proposed [22,23]. This method could reduce the gap between slices, offering a substantial decreased scan time, and alternatively, the saved time can be used to improve spatial resolution [24].

Previous studies on breast DWI have shown that SMS rs-EPI with an acceleration factor of 2 can markedly reduce scan time while maintaining similar SNR, ADC value and image quality compared with ss-EPI [25,26]. Nevertheless, the voxel size of DWI above is still much larger than that in DCE-MRI, and few reports exist concerning applications of SMS technique in improving spatial resolution. Improving of image resolution could more accurately characterize the morphology of breast lesions and clearly show the margin and internal heterogeneity of the lesion, which are important for distinguishing features of malignant tumors. Thus, the purpose of this work was to evaluate the feasibility of high-resolution SMS rs-EPI for the assessment of malignant and benign breast lesions.

## 2. Materials and Methods

### 2.1. Study Patients

This study was approved by the institutional ethics committee. From August 2019 to December 2019, a total of 374 patients with suspected breast lesions underwent breast MRI examinations in our department. After reading the informed consent, patients who did not wish to undergo SMS rs-EPI DWI sequences were excluded (*n* = 178); thus, 196 patients underwent prototype SMS rs-EPI sequence in addition to the standard breast MRI protocol (including conventional rs-EPI sequence) scans. The patients with no abnormal enhancement or obvious lesion found in the breast (*n* = 67), breast implants (*n* = 16) or obvious motion artifacts (*n* = 11) were excluded. Finally, a total of 102 patients with 113 lesions (54 of malignant, 59 of benign) were included in the final analysis.

### 2.2. MR Protocol

All examinations were performed on a 3T MRI scanner with a dedicated 18-channel phased-array breast coil (MAGNETOM Skyra, Siemens Healthcare, Erlangen, Germany). The patient was in prone position with head first for the scan.

The standard MR imaging examination consisted of the following protocol: T2 turbo inversion recovery magnitude (TR 4300 ms, TE 61.0 ms, section thickness 4 mm, FOV 340 × 340 mm); axial 3D fast low angle shot (FLASH) T1 (TR 6.05 ms, TE 2.46 ms, section thickness 1.3 mm, FOV 340 × 340 mm); T2-weighted turbo spin-echo (TR 4500 ms, TE 79 ms, section thickness 4.0 mm, FOV 340 × 340 mm). DCE imaging was performed with one pre-contrast and five post-contrast dynamic series using axial 3D FLASH T1 with fat suppression (TR 4.67 ms, TE 1.66 ms, section thickness 1.3 mm, FOV 360 × 360 mm). A 0.1-mmol/kg bolus of Gadobenate (Multihance, BRACCO, Milano) was injected using high pressure injector, followed by a 15 mL saline flush.

For DWI sequences, conventional rs-EPI was obtained, and then prototype SMS rs-EPI DWI sequences were applied. Both DWI sequences were acquired after T2 but prior to DCE, followed by SMS rs-EPI. We chose the highest resolution that can be achieved in the acquisition time of 200 s for the experiment, such that the scan time does not increase beyond what is clinically acceptable. The details of imaging parameters of conventional rs-EPI and SMS rs-EPI sequence are shown in Table 1.

### 2.3. Image Analysis

#### 2.3.1. Image Quality

All imaging datasets were independently assessed by two radiologists (F.Y. and J.T., with 26 and 18 years of experience which specialized on breast MR imaging, respectively). The readers were blinded to the sequence and the histopathology results. During the reading procedure, the images of two DWI sequences (conventional rs-EPI and SMS rs-EPI) were evaluated together. Image quality was assessed on the b = 1000 s/mm^2^ images and based on a 5-point Likert scale with respect to the following categories [26,27,28]:

- Anatomic distortion (1 = very strong, 2 = strong, 3 = medium, 4 = small, 5 = negligible);
- Lesion conspicuity (1 = not diagnostic, 2 = poor, 3 = moderate, 4 = good, 5 = excellent);
- Sharpness of anatomical details (1 = not diagnostic, 2 = poor, 3 = moderate, 4 = good, 5 = excellent);
- Overall image quality (1 = not diagnostic, 2 = poor, 3 = moderate, 4 = good, 5 = excellent).

#### 2.3.2. Lesion Size

For lesion size, the measurement was performed on DCE sequence (the late phase of enhancement), the largest long diameter of tumor was measured in long-axis using the slice on which the lesion appeared largest.

#### 2.3.3. Signal-to-Noise Ratio

**Figure 1:**
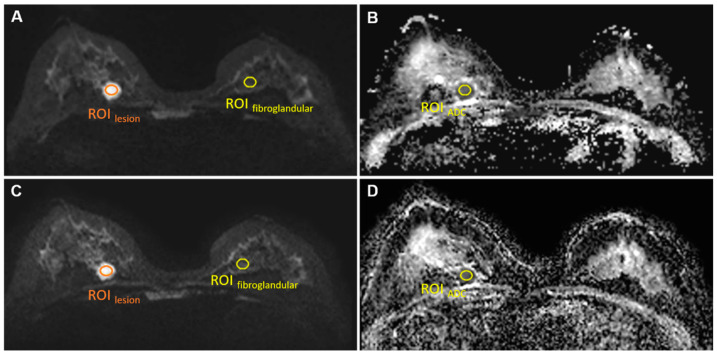
ROI_lesion_, ROI_fibroglandular_ on b = 1000 s/mm^2^ images conventional rs-EPI (**A**) and SMS rs-EPI (**C**); ROI_ADC_ on ADC map of conventional rs-EPI (**B**) and SMS rs-EPI (**D**).

For both sequences, a single region of interest (ROI_lesion_, size 30–50 mm^2^; Figure 1) was manually drawn by two radiologists (S.P. and L.C., with 5 and 3 years of experience) on the slice with the largest cross section of the lesion on the b = 1000 s/mm^2^ images for the signal intensity measurement of lesions (SI_lesion_). In addition, another ROI (size 50 mm^2^) was drawn manually in the anterior area outside of the breast to measure background noise signal intensity (SI_noise_).

The signal-to-noise ratio (SNR) was defined as follows [29,30]:

$$\mathrm{SNR}\;=\frac{{\mathrm{SI}}_{\mathrm{lesion}}}{{\mathrm{SI}}_{\mathrm{noise}}}$$

#### 2.3.4. Lesion Contrast-to-Noise Ratio

The lesion contrast-to-noise ratio (CNR) was defined as follows [30]:

$$\mathrm{CNR}\;=\frac{{\mathrm{SI}}_{\mathrm{lesion}}-{\mathrm{SI}}_{\mathrm{contralateral}\;\mathrm{fibroglandular}}}{{\mathrm{SD}}_{\mathrm{noise}}}$$

Here, SI_lesion_ was measured using the same ROI_lesion_ as in the SNR measurements. The same ROI was copied to normal contralateral fibroglandular tissue for normal breast tissue signal intensity (${\mathrm{SI}}_{\mathrm{contralateral}\;\mathrm{fibroglandular}}$) measurement on the same slice in b = 1000 s/mm^2^ image (Figure 1). ${\mathrm{SD}}_{\mathrm{noise}}$ is the standard deviation of the background noise signal intensity.

#### 2.3.5. ADC Value

The ADC value was measured using the same ROI of the lesion as in the SNR measurements (Figure 1). The copy-and-paste function of the post-processing software was used to place the ROI on the corresponding ADC maps.

### 2.4. Statistics

The data were statistically analyzed using SPSS 21.0 software (IBM Corp, Armonk, NY, USA). The Likert scales of image quality were compared using the Wilcoxon signed-rank test. The paired t test was used for the quantitative evaluation of SNR and CNR. For the comparison of the ADC value, two-way analysis of variance (ANOVA) was performed. A *p* value less than 0.05 was considered statistically significant. Inter-reader agreement of quantitative measurements (SNR, CNR and ADC value) and qualitative image score were assessed by calculating respective intraclass correlation coefficients (ICC) and Cohen’s kappa, respectively [31,32].

The receiver operating characteristic (ROC) curve analysis was performed to evaluate the diagnostic performance of the ADC value. The cutoff value, sensitivity, and specificity of ROC curve were determined by using the maximum Youden index (sensitivity + specificity-1) [33].

## 3. Results

### 3.1. Patients and Lesions

Breast MRI was successfully performed in 102 patients (mean age 42.57 ± 12.33 years), and a total of 113 lesions were identified with 54 malignant lesions (mean age 47.96 ± 11.22 years) and 59 (mean age 37.63 ± 11.24 years) benign lesions. Malignant lesions confirmed by pathology were composed of 47 invasive ductal carcinomas (IDC), four ductal carcinomas in situ (DCIS), one invasive lobular carcinoma (ILC) and two mucinous carcinomas. Within the benign lesions, 37 of 59 were histopathologically confirmed by biopsy, including 22 fibroadenomas, seven fibroadenomas with mucinous degeneration, four phyllodes tumor, one fibrocystic change and three adenosis. In the remaining 22 lesions, a biopsy was not available because the lesions were clearly radiologically benign and was defined as long term imaging and clinical follow up. The largest long diameter was 17.85 ± 8.27 mm for all malignant lesions and 11.43 ± 5.36 mm for all benign lesions (*p* = 0.046).

### 3.2. Comparison of Image Quality

**Figure 2:**
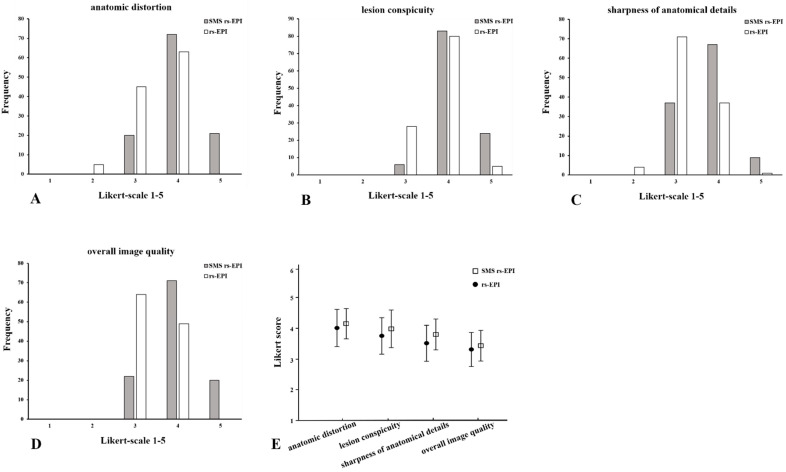
Histogram of the evaluation of image quality: anatomic distortion (**A**), lesion conspicuity (**B**), sharpness of anatomical details (**C**) and overall image quality (**D**) using Likert scoring; mean values and standard deviation of quality scores (**E**). Comparison of SMS rs-EPI sequence and rs-EPI sequence.

The image scores between the reviewers in anatomic distortion, lesion conspicuity, sharpness of anatomical details and overall image quality of SMS rs-EPI were significantly higher than those of conventional rs-EPI (*p* < 0.001) (Table 2 and Figure 2). The average scores of two reviewers for SMS rs-EPI vs. conventional rs-EPI were as follows: anatomic distortion 3.96 ± 0.56 vs. 3.48 ± 0.56, lesion conspicuity 4.12 ± 0.47 vs. 3.81 ± 0.46, sharpness of anatomical details 3.73 ± 0.58 vs. 3.27 ± 0.50, and overall image quality 4.00 ± 0.60 vs. 3.42 ± 0.49, *p* < 0.001.).

**Figure 3:**
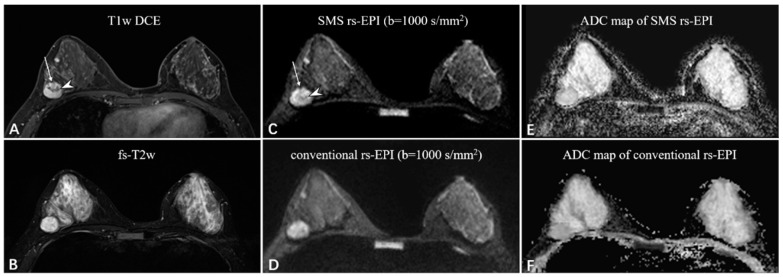
A 38-year-old woman with fibroadenoma in the right breast. The oval mass with slight lobulation (long white arrow) shows heterogeneous enhancement with dark internal septation (white arrowhead) on the T1-weighted DCE images (**A**) and strong high-signal intensity on the fat-saturated T2-weighted images (**B**). Compared to the rs-EPI image (**D**), the SMS rs-EPI image (**C**) shows better sharpness of anatomical details, in which the lobulated margin pointed by long white arrows and internal heterogeneous component pointed by white arrowheads are clearly shown. The lesion is more conspicuous with a clear margin and less distortion in the ADC map of SMS rs-EPI, although there is no ADC reduction due to the benign nature of the lesion (**E**,**F**).

**Figure 4:**
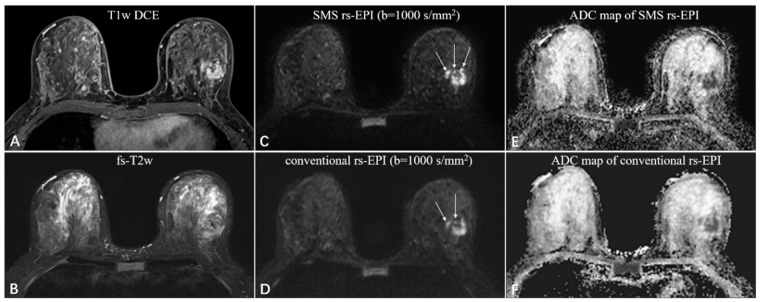
A 46-year-old woman with invasive ductal carcinoma in the left breast. The irregular mass with heterogeneous enhancement and multiple small foci around on the T1-weighted DCE images (**A**), and equal-to-low signal intensity on the fat-saturated T2-weighted images (**B**). The SMS rs-EPI image (**C**) shows better sharpness of anatomical details (pointed by long white arrows) and less anatomic distortion than rs-EPI image (**D**). The ADC signal is obviously decreased in both sequences (**E**,**F**).

Compared to conventional rs-EPI, the scan time of SMS rs-EPI sequence with higher spatial resolution did not increase (200 s vs. 224 s), which is listed in Table 1. SNR and CNR assessment are shown in Table 3. The SNR was not different between the two sequences (7.54 ± 2.15 vs. 7.41 ± 2.47; *p* = 0.352), while CNR was higher in high-resolution SMS rs-EPI sequence (6.48 ± 1.71 vs. 4.23 ± 1.49; *p* < 0.001; Figure 3 and Figure 4).

The inter-reader agreement for all image scores ranged between fair and excellent agreement (Cohen’s kappa = 0.558–0.713). The inter-observer agreement was excellent for SNR (ICC = 0.719 (0.617–0.798) and 0.751 (0.658–0.821)) and good for CNR (ICC = 0.627 (0.499–0.728) and 0.669 (0.553–0.759)) in both sequences.

The figures (Figure 3 and Figure 4) showed representative images of benign and malignant lesions. A 38-year-old woman with fibroadenoma in the right breast was shown in Figure 3. A 46-year-old woman with invasive ductal carcinoma in the left breast is shown in Figure 4.

### 3.3. Comparison of ADC Value

**Figure 5:**
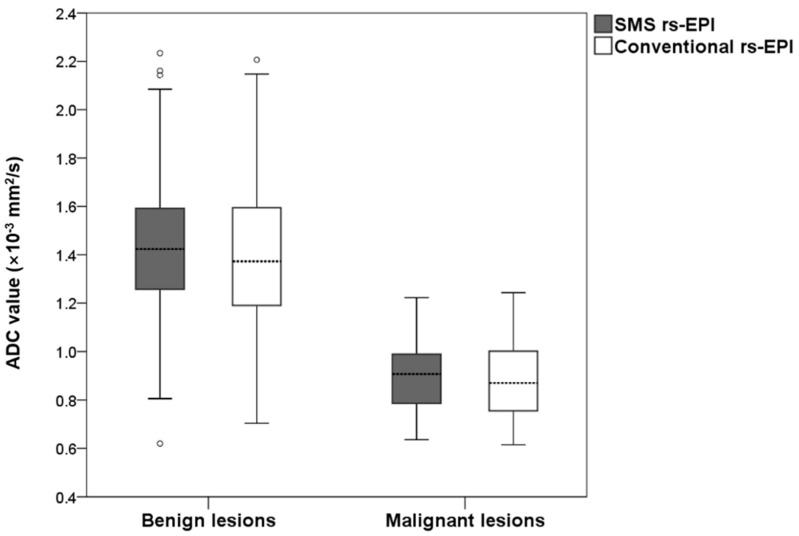
Boxplot of the ADC values of malignant and benign lesions for SMS rs-EPI and conventional rs-EPI.

The measured ADC values for benign and malignant lesions are shown in Figure 5. The mean ADC of benign lesions in SMS rs-EPI was equal to those in conventional rs-EPI (1.43 × 10^−3^ mm^2^/s vs. 1.43 × 10^−3^ mm^2^/s, *p* = 0.717). The mean ADC of malignant lesions in SMS rs-EPI was the same as that in conventional rs-EPI (0.91 × 10^−3^ mm^2^/s vs. 0.89 × 10^−3^ mm^2^/s, *p* = 0.680). The inter-observer agreement was good for ADC values in both SMS rs-EPI sequences and conventional rs-EPI sequences (ICC = 0.861 (0.805–0.902) and 0.718 (0.615–0.797)).

**Figure 6:**
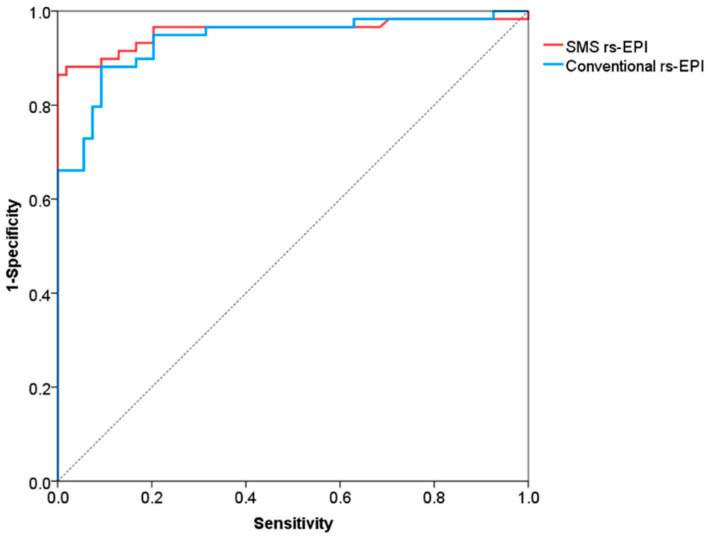
Receiver operating characteristic curves for differentiation of malignant and benign lesions.

The ROC curves for differentiation of malignant and benign breast lesions in SMS rs-EPI and conventional rs-EPI are shown in Figure 6 and its corresponding AUC, cutoff, sensitivity and specificity are listed in Table 4. The AUCs of SMS rs-EPI and conventional rs-EPI were 0.957 (95%CI, 0.916–0.999; sensitivity, 0.86; specificity, 1.00) and 0.938 (95%CI, 0.894–0.983; sensitivity, 0.88; specificity, 0.91) (Table 4). The cutoff value of ADC to distinguish malignant and benign lesions was 1.22 × 10^−3^ mm^2^/s (sensitivity = 0.86; specificity = 1.00) for SMS rs-EPI and 1.14 × 10^−3^ mm^2^/s (sensitivity = 0.88; specificity = 0.91) for rs-EPI (Table 4).

## 4. Discussion

With the development of the rs-EPI sequence, the sensitivity for detecting breast lesion was much improved due to its high contrast between tumors and healthy tissues [13,34]. Compared to ss-EPI, rs-EPI based on the segmentation of k-space can shorten the echo spacing and the echo train duration, thereby reducing phase-encoding distortion artifacts and T2* blurring [21,35]. Previous studies demonstrated that the use of rs-EPI sequence can significantly improve the overall image quality and lesion conspicuity of breast DWI [17,21,34,36]; however, compared to ss-EPI, the rs-EPI sequence requires a longer acquisition time. In this study, the result revealed that the SMS acquisition technique can not only offer a decreased scan time of rs-EPI sequence but can also display higher image quality compared with rs-EPI.

Different from the standard parallel imaging, SMS leads to a reduction in acquisition time of the EPI readout, with no impact on distortion or SNR [22]. Theoretically, slice acceleration decreases the SNR because of TR shortening and a growing g-factor penalty [37]; however, a previous study has shown that SNR efficiency is expected to increase with slice acceleration for shorter TR [23,25]. Thus, in our study, no significant difference in SNR was observed between two sequences, while the CNR of SMS rs-EPI was significantly higher than that of conventional rs-EPI. This indicated that the SMS technique may improve lesion display conspicuity, which can increase the clinical use of rs-EPI DWI for assessing breast lesion.

A previous study [26] that evaluated the diagnostic value of SMS-EPI in breast lesions showed that the mean ADC values for benign lesions were 1.86 × 10^−3^ mm^2^/s (rs-EPI) and 1.86 × 10^−3^ mm^2^/s (SMS-EPI), and for malignant lesions were 0.90 × 10^−3^ mm^2^/s (rs-EPI) and 0.89 × 10^−3^ mm^2^/s (SMS-EPI). Our results showed similar ADC values for malignant lesions and inferior ADC values for benign lesions. This is possibly because our study did not include cystic lesions and fibrocystic changes. Moreover, our results showed that no significant deviations of ADC values were observed between SMS rs-EPI and conventional rs-EPI in the breast, which is consistent with several recently published studies [25,26,28,38,39]. The use of SMS technology in our research has no effect on ADC measurement.

SMS technology in previous studies is mostly used to reduce the acquisition time of breast DW images based on rs-EPI. The spatial resolution of breast DWI is usually about 4–15.625 mm^3^ [25,26,28,39,40,41], which is much lower than the spatial resolution of DCE-MRI (less than 1 mm^3^) [42]. The larger voxel size isotropic sequence of DCE-MRI was superior in terms of lesion detection, which can reduce the occurrence of partial volume effects, clearly showing the margin of the lesion and the detection of tiny lesions [43,44]. However, limited by acquisition time, conventional rs-EPI showed relatively low resolution, leading to challenges in the evaluation of intra-tumor heterogeneity and differentiation between tumor and normal glandular tissue. In this study, we used the time saved with SMS acceleration technique for higher in-plane resolution and slice resolution. Compared with the conventional rs-EPI, we increased the resolution from 1.5 × 1.5 × 4.0 mm^3^ to 1.3 × 1.3 × 3.0 mm^3^ with SMS rs-EPI, and still offered a reduction of overall acquisition time by 24 s. The high spatial resolution DWI with SMS acceleration can help increase the conspicuity of lesions and the sharpness of anatomical details and describe the shape and margins of the breast lesions accurately, which is also the differentiating criteria between benign and malignant lesions in BI-RADS [4]. We found more small lesions (diameter range 1–3 mm) detected in nine patients when evaluated with the SMS rs-EPI sequence instead of the conventional rs-EPI sequence, which is of great help to the surgeon in determining the scope of surgery. These encouraging results demonstrated the feasibility of high spatial resolution DWI with SMS acceleration in the evaluation of breast lesions and indicated its potential in routine clinical practice.

This study has several limitations. First, only two-fold SMS acquisition was compared to rs-EPI; the differences among multiple SMS-accelerated factor setups were not considered. Second, all images were acquired on a 3T MR system; the application of SMS rs-EPI in different field strengths remains to be explored. Third, the SNR was calculated by dividing the average signal intensity measurement of lesions by the background noise signal intensity in this study; however, the background of an accelerated image does not have a uniform noise distribution; using the background noise signal intensity to calculate the SNR may cause artificial deviations [45]. Therefore, the method of SNR measurement should be improved in future research. Finally, compared with malignant lesions, the diameter of most benign lesions is relatively small, which will cause some deviations in the measured values.

## 5. Conclusions

SMS rs-EPI allows for higher spatial resolution and a slight reduction of scan time in comparison to conventional rs-EPI.

## Figures and Tables

**Table 1:** Imaging parameters of SMS rs-EPI sequence and conventional rs-EPI.

Sequence	SMS rs-EPI	Conventional rs-EPI
orientation	transversal	transversal
fat suppressed	SPAIR	SPAIR
TR (ms)	4550	6730
TE (ms)	58	67
voxel size (mm)	1.3 × 1.3 × 3.0	1.5 × 1.5 × 4.0
FOV (mm^2^)	170 × 280	170 × 280
imaging matrix	131 × 214	114 × 188
bandwidth (Hz/Px)	835	885
slice acceleration factor	2	-
GRAPPA acceleration factor	2	2
b values, s/mm^2^	50, 1000	50, 1000
acquisition time (s)	200	224
number of images	42	30

SPAIR, spectral attenuated inversion recovery; TR, repetition time; TE, echo time; FOV, field of view; GRAPPA, generalized autocalibrating partially parallel acquisitions.

**Table 2:** Subjective image quality assessment of SMS rs-EPI and conventional rs-EPI.

		SMS rs-EPI	rs-EPI	*p* Value
anatomic distortion	R1	4.01 ± 0.60	3.51 ± 0.58	
	R2	3.92 ± 0.50	3.44 ± 0.53	
	R1 + R2 (average)	3.96 ± 0.56	3.48 ± 0.56	<0.001
lesion conspicuity	R1	4.16 ± 0.49	3.80 ± 0.50	
	R2	4.09 ± 0.45	3.83 ± 0.42	
	R1 + R2 (average)	4.12 ± 0.47	3.82 ± 0.46	<0.001
sharpness of anatomical details	R1	3.75 ± 0.59	3.31 ± 0.55	
	R2	3.71 ± 0.58	3.24 ± 0.45	
	R1 + R2 (average)	3.73 ± 0.58	3.27 ± 0.50	<0.001
overall image quality	R1	3.98 ± 0.61	3.43 ± 0.50	
	R2	4.03 ± 0.59	3.42 ± 0.50	
	R1 + R2 (average)	4.00 ± 0.60	3.42 ± 0.49	<0.001

R1/R2, reader one and two. *p* values of less than 0.05 were considered to show statistical significance.

**Table 3:** Objective image quality assessment of SMS rs-EPI and conventional rs-EPI.

		SMS rs-EPI	rs-EPI	*p* Value
SNR	R1	7.67 ± 2.24	7.40 ± 2.61	
	R2	7.42 ± 2.39	7.42 ± 2.39	
	R1 + R2 (average)	7.54 ± 2.15	7.41 ± 2.47	0.352
CNR	R1	6.31 ± 1.74	4.27 ± 1.68	
	R2	6.67 ± 2.05	4.18 ± 2.05	
	R1 + R2 (average)	6.48 ± 1.71	4.23 ± 1.49	<0.001

SNR, signal-to-noise ratio; CNR, contrast-to-noise ratio. *p* values less than 0.05 were considered to show statistical significance.

**Table 4:** Diagnostic efficiency of ADC value in SMS rs-EPI and conventional rs-EPI.

	AUC (95%CI)	Sensitivity	Specificity	Cutoff Value (×10^−3^ mm^2^/s)
SMS rs-EPI	0.957 (0.916–0.999)	0.86	1.00	1.22
rs-EPI	0.938 (0.894–0.983)	0.88	0.91	1.14

## Data Availability

The datasets generated during and/or analyzed during the current study are available from the corresponding author on reasonable request.

## References

[1] Siegel R.L., Miller K.D., Jemal A. (2019). Cancer statistics, 2019. CA Cancer J. Clin..

[2] Berg W.A., Gutierrez L., NessAiver M.S., Carter W.B., Bhargavan M., Lewis R.S., Ioffe O.B. (2004). Diagnostic accuracy of mammography, clinical examination, US, and MR imaging in preoperative assessment of breast cancer. Radiology.

[3] Fowler A.M., Mankoff D.A., Joe B.N. (2017). Imaging Neoadjuvant Therapy Response in Breast Cancer. Radiology.

[4] D’Orsi C.J., Sickles E.A., Mendelson E.B., Morris E.A. (2013). ACR BI-RADS^®^Atlas, Breast Imaging Reporting and Data System.

[5] Moon M., Cornfeld D., Weinreb J. (2009). Dynamic contrast-enhanced breast MR imaging. Magn. Reson. Imaging Clin. N. Am..

[6] Pinker K., Moy L., Sutton E.J., Mann R.M., Weber M., Thakur S.B., Jochelson M.S., Bago-Horvath Z., Morris E.A., Baltzer P.A.T. (2018). Diffusion-Weighted Imaging with Apparent Diffusion Coefficient Mapping for Breast Cancer Detection as a Stand-Alone Parameter: Comparison with Dynamic Contrast-Enhanced and Multiparametric Magnetic Resonance Imaging. Investig. Radiol..

[7] Pinker K., Grabner G., Bogner W., Gruber S., Trattnig S., Heinz-Peer G., Fitzal F., Pluschnig U., Rudas M., Helbich T. (2009). A combined high temporal and high spatial resolution 3 Tesla MR imaging protocol for the assessment of breast lesions: Initial results. Investig. Radiol..

[8] Pinker K., Helbich T.H., Morris E.A. (2017). The potential of multiparametric MRI of the breast. Br. J. Radiol..

[9] Zhang L., Tang M., Min Z., Lu J., Lei X., Zhang X. (2016). Accuracy of combined dynamic contrast-enhanced magnetic resonance imaging and diffusion-weighted imaging for breast cancer detection: A meta-analysis. Acta Radiol..

[10] Kul S., Cansu A., Alhan E., Dinc H., Gunes G., Reis A. (2011). Contribution of diffusion-weighted imaging to dynamic contrast-enhanced MRI in the characterization of breast tumors. AJR Am. J. Roentgenol..

[11] Malayeri A.A., El Khouli R.H., Zaheer A., Jacobs M.A., Corona-Villalobos C.P., Kamel I.R., Macura K.J. (2011). Principles and Applications of Diffusion-weighted Imaging in Cancer Detection, Staging, and Treatment Follow-up. Radiographics.

[12] Park M.J., Cha E.S., Kang B.J., Ihn Y.K., Baik J.H. (2007). The role of diffusion-weighted imaging and the apparent diffusion coefficient (ADC) values for breast tumors. Korean J. Radiol..

[13] Min Q., Shao K., Zhai L., Liu W., Zhu C., Yuan L., Yang J. (2015). Differential diagnosis of benign and malignant breast masses using diffusion-weighted magnetic resonance imaging. World J. Surg. Oncol..

[14] Santamaria G., Bargallo X., Fernandez P.L., Farrus B., Caparros X., Velasco M. (2017). Neoadjuvant Systemic Therapy in Breast Cancer: Association of Contrast-enhanced MR Imaging Findings, Diffusion-weighted Imaging Findings, and Tumor Subtype with Tumor Response. Radiology.

[15] Park S.H., Moon W.K., Cho N., Song I.-C., Chang J.M., Park I.-A., Han W., Noh D.-Y. (2010). Diffusion-weighted MR imaging: Pretreatment prediction of response to neoadjuvant chemotherapy in patients with breast cancer. Radiology.

[16] Ramirez-Galvan Y.A., Cardona-Huerta S., Elizondo-Riojas G., Alvarez-Villalobos N.A. (2018). Apparent Diffusion Coefficient Value to Evaluate Tumor Response after Neoadjuvant Chemotherapy in Patients with Breast Cancer. Acad Radiol..

[17] Porter D.A., Heidemann R.M. (2009). High resolution diffusion-weighted imaging using readout-segmented echo-planar imaging, parallel imaging and a two-dimensional navigator-based reacquisition. Magn. Reson. Med..

[18] Skare S., Newbould R.D., Clayton D.B., Albers G.W., Nagle S., Bammer R. (2007). Clinical multishot DW-EPI through parallel imaging with considerations of susceptibility, motion, and noise. Magn. Reson. Med..

[19] Li H., Liu L., Shi Q., Stemmer A., Zeng H., Li Y., Zhang M. (2017). Bladder cancer: Detection and image quality compared among iShim, RESOLVE, and ss-EPI diffusion-weighted MR imaging with high b value at 3.0 T MRI. Medicine.

[20] Kishimoto A.O., Kataoka M., Iima M., Honda M., Miyake K.K., Ohashi A., Ota R., Kataoka T., Sakurai T., Toi M. (2020). The comparison of high-resolution diffusion weighted imaging (DWI) with high-resolution contrast-enhanced MRI in the evaluation of breast cancers. Magn. Reson. Imaging.

[21] Bogner W., Pinker-Domenig K., Bickel H., Chmelik M., Weber M., Helbich T.H., Trattnig S., Gruber S. (2012). Readout-segmented echo-planar imaging improves the diagnostic performance of diffusion-weighted MR breast examinations at 3.0 T. Radiology.

[22] Barth M., Breuer F., Koopmans P.J., Norris D.G., Poser B.A. (2016). Simultaneous multislice (SMS) imaging techniques. Magn. Reson. Med..

[23] Setsompop K., Gagoski B.A., Polimeni J.R., Witzel T., Wedeen V.J., Wald L.L. (2012). Blipped-controlled aliasing in parallel imaging for simultaneous multislice echo planar imaging with reduced g-factor penalty. Magn. Reson. Med..

[24] Taron J., Martirosian P., Kuestner T., Schwenzer N.F., Othman A., Weiß J., Notohamiprodjo M., Nikolaou K., Schraml C. (2018). Scan time reduction in diffusion-weighted imaging of the pancreas using a simultaneous multislice technique with different acceleration factors: How fast can we go?. Eur. Radiol..

[25] Filli L., Ghafoor S., Kenkel D., Liu W., Weiland E., Andreisek G., Frauenfelder T., Runge V.M., Boss A. (2016). Simultaneous multi-slice readout-segmented echo planar imaging for accelerated diffusion-weighted imaging of the breast. Eur. J. Radiol..

[26] Ohlmeyer S., Laun F.B., Palm T., Janka R., Weiland E., Uder M., Wenkel E. (2019). Simultaneous Multislice Echo Planar Imaging for Accelerated Diffusion-Weighted Imaging of Malignant and Benign Breast Lesions. Investig. Radiol..

[27] Boss A., Barth B., Filli L., Kenkel D., Wurnig M.C., Piccirelli M., Reiner C.S. (2016). Simultaneous multi-slice echo planar diffusion weighted imaging of the liver and the pancreas: Optimization of signal-to-noise ratio and acquisition time and application to intravoxel incoherent motion analysis. Eur. J. Radiol..

[28] Taron J., Martirosian P., Erb M., Kuestner T., Schwenzer N.F., Schmidt H., Honndorf V.S., Weiß J., Notohamiprodjo M., Nikolaou K. (2016). Simultaneous multislice diffusion-weighted MRI of the liver: Analysis of different breathing schemes in comparison to standard sequences. J. Magn. Reson. Imaging.

[29] Dietrich O., Raya J.G., Reeder S.B., Reiser M.F., Schoenberg S.O. (2007). Measurement of signal-to-noise ratios in MR images: Influence of multichannel coils, parallel imaging, and reconstruction filters. J. Magn. Reson. Imaging.

[30] Bickel H., Polanec S.H., Wengert G., Pinker K., Bogner W., Helbich T.H., Baltzer P.A. (2019). Diffusion-Weighted MRI of Breast Cancer: Improved Lesion Visibility and Image Quality Using Synthetic b-Values. J. Magn. Reson. Imaging.

[31] Landis J.R., Koch G.G. (1977). The measurement of observer agreement for categorical data. Biometrics.

[32] Bartko J.J. (1966). The intraclass correlation coefficient as a measure of reliability. Psychol. Rep..

[33] Youden W.J. (1950). Index for rating diagnostic tests. Cancer-Am. Cancer Soc..

[34] Hirano M., Satake H., Ishigaki S., Ikeda M., Kawai H., Naganawa S. (2012). Diffusion-weighted imaging of breast masses: Comparison of diagnostic performance using various apparent diffusion coefficient parameters. AJR Am. J. Roentgenol..

[35] Holdsworth S.J., Skare S., Newbould R.D., Bammer R. (2009). Robust GRAPPA-accelerated diffusion-weighted readout-segmented (RS)-EPI. Magn. Reson. Med..

[36] Kim Y.J., Kim S.H., Kang B.J., Park C.S., Kim H.S., Son Y.H., Porter D., Song B.J. (2014). Readout-segmented echo-planar imaging in diffusion-weighted mr imaging in breast cancer: Comparison with single-shot echo-planar imaging in image quality. Korean J. Radiol..

[37] Tu C., Shen H., Liu D., Chen Q., Yuan X., Li X., Wang X., Liu R., Wang X., Li Q. (2021). Simultaneous multi-slice readout-segmentation of long variable echo-trains for accelerated diffusion-weighted imaging of nasopharyngeal carcinoma: A feasibility and optimization study. Clin. Imaging.

[38] Taron J., Schraml C., Pfannenberg C., Reimold M., Schwenzer N., Nikolaou K., Martirosian P., Seith F. (2018). Simultaneous multislice diffusion-weighted imaging in whole-body positron emission tomography/magnetic resonance imaging for multiparametric examination in oncological patients. Eur. Radiol..

[39] Weiss J., Martirosian P., Taron J., Othman A.E., Kuestner T., Erb M., Bedke J., Bamberg F., Nikolaou K., Notohamiprodjo M. (2017). Feasibility of accelerated simultaneous multislice diffusion-weighted MRI of the prostate. J. Magn. Reson. Imaging.

[40] Machida Y., Nomura K., Shimauchi A., Kato Y., Nagatsuka M., Fukuma E. (2020). Diffusion-weighted imaging with simultaneous multi-slice echo-planar technique for the diagnosis of breast magnetic resonance imaging. Jpn. J. Radiol..

[41] Taviani V., Alley M.T., Banerjee S., Nishimura D.G., Daniel B.L., Vasanawala S.S., Hargreaves B.A. (2017). High-resolution diffusion-weighted imaging of the breast with multiband 2D radiofrequency pulses and a generalized parallel imaging reconstruction. Magn. Reson. Med..

[42] Delbany M., Bustin A., Poujol J., Thomassin-Naggara I., Felblinger J., Vuissoz P.A., Odille F. (2019). One-millimeter isotropic breast diffusion-weighted imaging: Evaluation of a superresolution strategy in terms of signal-to-noise ratio, sharpness and apparent diffusion coefficient. Magn. Reson. Med..

[43] Pinker K., Bogner W., Baltzer P., Trattnig S., Gruber S., Abeyakoon O., Bernathova M., Zaric O., Dubsky P., Bago-Horvath Z. (2014). Clinical application of bilateral high temporal and spatial resolution dynamic contrast-enhanced magnetic resonance imaging of the breast at 7 T. Eur. Radiol..

[44] Gruber S., Pinker K., Zaric O., Minarikova L., Chmelik M., Baltzer P., Boubela R.N., Helbich T., Bogner W., Trattnig S. (2014). Dynamic contrast-enhanced magnetic resonance imaging of breast tumors at 3 and 7 T: A comparison. Investig. Radiol..

[45] Altahawi F.F., Blount K.J., Morley N.P., Raithel E., Omar I.M. (2017). Comparing an accelerated 3D fast spin-echo sequence (CS-SPACE) for knee 3-T magnetic resonance imaging with traditional 3D fast spin-echo (SPACE) and routine 2D sequences. Skelet. Radiol..

